# Landscape of Monoclonal Antibodies Targeting Zika and Dengue: Therapeutic Solutions and Critical Insights for Vaccine Development

**DOI:** 10.3389/fimmu.2020.621043

**Published:** 2021-02-04

**Authors:** Vincent Dussupt, Kayvon Modjarrad, Shelly J. Krebs

**Affiliations:** ^1^ Emerging Infectious Diseases Branch, Walter Reed Army Institute of Research, Silver Spring, MD, United States; ^2^ U.S. Military HIV Research Program, Walter Reed Army Institute of Research, Silver Spring, MD, United States; ^3^ Henry M. Jackson Foundation for the Advancement of Military Medicine, Bethesda, MD, United States

**Keywords:** Zika virus, Dengue virus, neutralizing antibody, monoclonal antibody, vaccine, therapeutics

## Abstract

The unprecedented 2015–2016 Zika outbreak in the Americas sparked global concern and drove the rapid deployment of vaccine and therapeutic countermeasures against this re-emerging pathogen. Alongside vaccine development, a number of potent neutralizing antibodies against Zika and related flaviviruses have been identified in recent years. High-throughput antibody isolation approaches have contributed to a better understanding of the B cell responses elicited following infection and/or vaccination. Structure-based approaches have illuminated species-specific and cross-protective epitopes of therapeutic value. This review will highlight previously described monoclonal antibodies with the best therapeutic potential against ZIKV and related flaviviruses, and discuss their implications for the rational design of better vaccine strategies.

## Introduction

The first generation of flavivirus vaccines, such as the yellow fever virus (YFV) 17D vaccine, were developed empirically by serially passaging pathogenic virus in animal tissues until attenuated virus variants emerged that were suitable to be used as a safe, live and effective vaccine (1, 2). Others, like the licensed Japanese encephalitis virus (JEV) and tick-borne encephalitis virus vaccines are made from whole inactivated viral particles (3, 4), a strategy that has been recapitulated for experimental vaccines for other flaviviruses, such as dengue virus (DENV) and Zika virus (ZIKV) (5–7). In recent decades, the advent of a novel suite of technologies has enabled a more rational and targeted approach to the rapid discovery and development of immunogens for emerging pathogens (8, 9). In particular, whole genome sequencing has revolutionized vaccinology, translating genomic information to vaccine candidates, through a process termed “reverse vaccinology”. The next phase of this once fresh approach—known as “reverse vaccinology 2.0”—now assembles a more multi-faceted, seamless pipeline of complementary disciplines that 1) pre-screens human donors 2) identifies and sequences B cell receptors of highly specific or broadly reactive B cells 3) expresses these sequences as monoclonal antibodies (mAbs) and 4) structurally and functionally characterizes the targeted antibody epitopes. However, this pathway not only applies to the development of vaccines immunogens, but is now the basis to rapidly scale prophylactic and therapeutic monoclonal antibodies as well (10, 11). These new technologies were first pioneered through research of viral pathogens, such as HIV and influenza, for which effective vaccines traditionally have been difficult to develop (11–13). The biology and methodologies elucidated by working on these other viruses have paid dividends for the development of countermeasures to other infectious diseases, particularly flaviviruses, which threaten the health of people throughout the world. Over the last several years, a large number of monoclonal antibodies protective against flaviviruses have been reported, providing new insights into neutralizing epitopes, new avenues for vaccine design, and potential combinations of mAbs that may be used for cross-flavivirus prophylaxis or treatment. In this article, we will review the current status of neutralizing monoclonal antibodies against ZIKV and DENV and provide perspectives on protective epitopes that may be of therapeutic value or guide the development of successful flavivirus vaccines.

## The Complex Zika Virus/Dengue Virus Serologic Landscape

**Figure 1:**
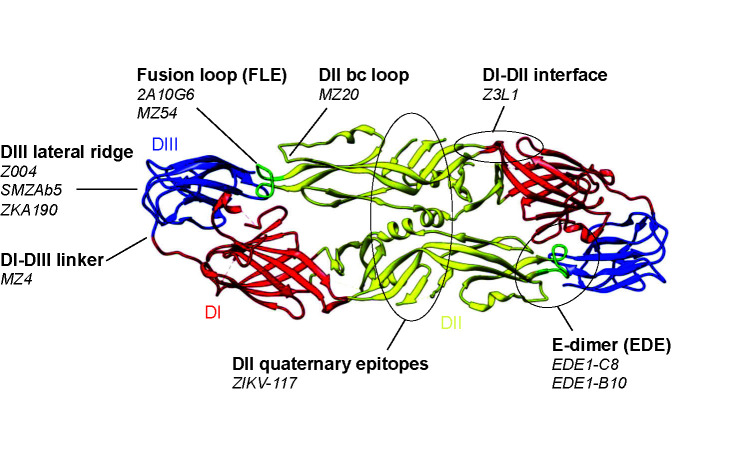
Neutralizing epitopes of Zika virus (ZIKV) and dengue virus (DENV) monoclonal antibodies. Top, Characteristics of prototypical ZIKV and DENV neutralizing antibodies grouped by epitope specificities. Neutralization potency is based on IC50 against ZIKV unless otherwise indicated. IC50 (ng/ml) are indicated as follows: **** (<10 ng/ml); *** (10–50 ng/ml); ** (50–500 ng/ml); * (>500 ng/ml). Bottom, the same neutralizing epitopes mapped onto the ZIKV E dimer. The ZIKV E dimer, PDB 5LBV (25), is shown in ribbon representation with the respective domains of each protomer colored in red (DI), yellow (DII), and blue (DIII) while the fusion loop in DII is highlighted in green. This visual was generated using UCSF Chimera (48).

Neutralizing antibody responses are the major correlate of protection from flavivirus infection (14–16). The primary target of these protective antibodies is the viral envelope (E) protein, incorporated into the budding virion as a premembrane (prM)-E precursor that is cleaved into membrane (M) and E proteins upon maturation. In mature particles, E is the only accessible protein at the virion surface, while M anchors E at the viral membrane. E consists of 3 domains DI, DII and DIII. The former, located at the center of the E protomer, links the putative host cell receptor binding domain, DIII, with DII, which drives dimerization and harbors the fusion loop at its distal end (**Figure 1**) (49). Most neutralizing antibodies act either by blocking virion binding to its host receptor or by interfering with the structural rearrangements necessary for E fusogenic activity, upon entry to the endocytic pathway (50). Mature ZIKV and DENV virions display 180 copies of E protein, arranged as anti-parallel dimers that cover the entire virion surface. These E dimers are further organized in an “herringbone” pattern with icosahedral architecture (51, 52). However, each of the three E proteins within an asymmetrical unit yields non-equivalent interactions. As a result, a given antibody epitope on the E monomer is presented in three different conformations on the mature particle. Quaternary epitopes, spanning several E protomers, are of great interest as they are often the target of potent neutralizing antibodies (22, 53). On the other hand, cryptic epitopes, transiently exposed due to the highly dynamic structure of E, have been associated with strain-specific poorly neutralizing responses (49).

**Table:** 

	Virus	Prototypical antibody	Similar antibodies	Species	Source, isolation strategy	Epitope,critical residues	Neutralizationpotency	Cross neutralization	References
**Fusion loop**	ZIKV-DENV	**2A10G6**	4G2	mouse	DENV-2 immunized mouse, hybridoma screening	Fusion loop W101	*	ZIKV, DENV1-4, WNV	(17–19)
	ZIKV-DENV	**MZ54**	MZ56	human	Flavivirus-experienced ZIKV vaccinee, B cell sort using whole virus + ZIKV/DENV E probes	Fusion loop W101	**	ZIKV, DENV1-4, WNV	(20)
**DI/DII**	ZIKV	**Z3L1**		human	ZIKV-infected human, memory B cell sort using ZIKV E probe	DI, DI-DII hinge	**	ZIKV specific	(21)
	DENV-1	**1F4**		human	B cells from DENV immune human, screening of EBV-transformed B cells from total PBMCs	DI, DI-DII hinge	***	DENV-1 specific	(22, 23)
**DII**	ZIKV-DENV	**MZ20**	1C19	human	Flavivirus-experienced ZIKV vaccinee, B cell sort using whole virus + ZIKV/DENV E probes	DII bc loop R73	**	ZIKV, DENV1-4, JEV	(20, 24)
**E dimer**	ZIKV/DENV	**EDE1-C8**	EDE1-B10, C10, EDE2-A11	human	Patients with acute DENV infection, screening of total plasmablasts	E dimer M68, S70, S72, G104, Q253, T315	*** (C8) **** (B10)	ZIKV, DENV1-4	(25–30)
	DENV-2	**2D22**		human	B cells from DENV immune human, screening of EBV-transformed B cells from total PBMCs	E dimer	** (DENV-2)	DENV-2 specific	(21, 31)
**DIII lateral ridge**	ZIKV	**ZV-67**	ZV-54	mouse	ZIKV-infected mouse with EDIII boost, hybridoma screening	DIII lateral ridge K394	**	ZIKV specific	(32)
	ZIKV/DENV-1	**Z004**	Z006, 1C11, ZIKV-116, SMZAb5	human	B cell from a ZIKV-infected human, memory B cell sort using ZIKV EDIII probe	DIII lateral ridge E393, K394	****	ZIKV, DENV-1	(33–37)
	DENV-1	**E105**	E106	mouse	DENV-1 infected mouse with EDIII boost, hybridoma screening	DIII lateral ridge	***	DENV-1 specific	(38)
	DENV-2	**3H5**	2C8	mouse	DENV-2 infected mouse, hybridoma screening	DIII lateral ridge	****	DENV-2 specific	(39, 40)
	ZIKV	**ZKA190**		human	B cell from a ZIKV-infected human, EBV-transformed B cell from total memory B cells	DI-DIII linker, DIII lateral ridge	***	ZIKV specific	(41, 42)
**DI/DIII linker**	ZIKV/DENV	**MZ4**	MZ1, MZ2, MZ24	human	Flavivirus-experienced ZIKV vaccinee, B cell sort using whole virus + ZIKV/DENV E probes	DI-DIII linker Y305	****	ZIKV, DENV1-4	(20)
	DENV-4	**5H2**		chimpanzee	Phage display from repertoire of infected chimpanzee	DI, DI-DIII linker	** (DENV-4)	DENV-4 specific	(43, 44)
**Quaternary**	ZIKV	**ZIKV-117**		human	B cell from a ZIKV-infected human, screening of EBV-transformed B cells from total PBMCs	dimer-dimer interface D67, Q89, K118	****	ZIKV specific	(45, 46)
	DENV-3	**5J7**		human	B cells from DENV immune human, screening of EBV-transformed B cells from total PBMCs	Quaternary DI-DII hinge, DII, DIII on 3 different protomers	****	DENV-3 specific	(22, 47)

Whereas E proteins across all flaviviruses share sequence and structural similarities, phylogenetic analyses have revealed that ZIKV is most related to the 4 DENV serotypes (25). Accordingly, both DENV convalescent plasma and DENV-directed monoclonal antibodies show substantial cross-reactivity to ZIKV (54, 55). These findings have prompted some investigators to consider ZIKV as the 5^th^ member of the DENV serocomplex (54). Investigations into the epidemiologic overlap of DENV and ZIKV, in areas where the two viruses co-circulate, have revealed important findings about their immunologic cross-reactivity. For example, pre-existing DENV immunity in humans was associated with reduced ZIKV symptoms in a pediatric cohort in Nicaragua (56), as well as reduced symptoms and a lower risk of ZIKV infection in a large prospective Brazilian cohort (57). Additionally, mothers who were infected with ZIKV, but had serologic evidence of pre-existing DENV, showed no increased risk of microcephaly in their fetuses (58). There are sufficient data to suggest serologic crosstalk between ZIKV and the four DENV serotypes that requires consideration when designing vaccines and antibody-based therapies.

## Neutralizing Antibodies Against Zika And Dengue

In an effort to develop categorical solutions to virus genuses or families, passive immunotherapy is gaining traction as a plausible alternative to vaccines and anti-virals. This has been made possible by the identification of highly potent and/or broadly cross-reactive antibodies (9), through large-scale single B cell isolation and screening approaches. These exceptionally potent antibodies have 50% inhibitory concentration (IC50) in the low ng/ml range, affording protection at concentrations readily achievable *in vivo*. The vast majority of these antibodies neutralize flaviviruses by locking E dimers in the pre-fusion conformation and preventing structural rearrangements necessary for E fusogenic activity. Engineering of these neutralizing antibodies can further enhance their potency and breadth as well as improve their pharmacokinetic properties (59, 60), so that a single infusion could confer protection over several weeks. Other modifications such as fine-tuning or abrogation of their effector functions are valuable tools (61, 62). However, given concerns of antibody-dependent enhancement (ADE) of infection between ZIKV and DENV (41, 54, 63, 64), ZIKV therapeutic mAbs would likely be best deployed harboring Fc-silenced mutations (LALA or YTE/FQQ) (65–68), to eliminate any risks of potentiating DENV infections, especially when Fc effector functions appear dispensable for protection in small animal models (26, 33, 41, 45).

### The E Dimer Epitope

A well-described protective epitope of flaviviruses is the E dimer epitope (EDE), a conserved quaternary epitope that is the target of potent cross-neutralizing antibodies. Originally discovered in DENV-infected patients (27), EDE mAbs were subsequently found to cross-neutralize ZIKV, and with high potency like EDE1-C8 (25) (**Figure 1**). The EDE is a particularly vulnerable site that is highly conserved across flaviviruses, as it overlaps with the binding site of prM in the immature particle. prM plays a key role during viral egress in the acidic Golgi compartment of the producer cell by preventing premature exposure of the fusion loop.

B10, another EDE mAb, was found to be particularly potent against ZIKV and protected mice against lethality and fetal demise (26). It provided robust therapeutic as well as prophylactic efficacy against ZIKV infection in rhesus monkeys and, importantly, did not induce viral escape, which is rare in antibody monotherapy (28).

The discovery of these potent EDE mAbs encouraged the development of immunogens capable of inducing B cell responses of similar specificities through vaccination. The addition of disulfide bonds necessary to stabilize the dimeric E also has the secondary benefit of masking the fusion loop epitope (69), another conserved epitope that has yielded cross-reactive but poorly neutralizing antibody responses (49). Stabilized dimers also present virus-specific, protective E dimer epitopes, recognized by mAbs such as 2D22 (70), a DENV-2 specific antibody. Efforts to develop EDE immunogens are ongoing and hold promise in eliciting broad and potent flavivirus protective responses (71).

### Domain III-Directed Antibodies

Antibodies to DIII have been shown to develop at later stages of ZIKV infection, and are associated with increased neutralization potency (72). While DIII antibodies have been readily elicited in murine models (32, 73) and are believed to be less frequent in humans (74), several potent DIII antibodies have been isolated from ZIKV-infected patients (33–37) (**Figure 1**). Prototypical DIII neutralizing antibodies target the lateral ridge epitope; these include mouse mAbs 3H5 (DENV-2) (40), E16 (WNV) (75), ZV-67 (ZIKV) (67) and human mAbs Z004 (34), SMZAb5 (33) and ZKA190 (42). Unlike the EDE directed B10 mAb, passive immunotherapy with single DIII antibodies quickly select for resistant virus variants. Therefore, a combination of at least two DIII antibodies (37) or the use of bi-specific antibodies targeting different domains (42) may prevent viral escape and are likely to be more effective for therapeutic use.

### DI/DIII Linker

A new class of potent ZIKV/DENV-2 cross-neutralizing antibodies targeting the DI/DIII linker region was recently identified in a donor with pre-existing DENV immunity following vaccination with a whole ZIKV inactivated virus. One prototypic mAb, MZ4, is highly potent against ZIKV and DENV-2 (20) (**Figure 1**). Since the DI/DIII linker is highly conserved across flavivirus species, secondary contacts with DI and DIII likely define the breadth and potency of such antibodies. Other mAbs such as ZKA190 (42) and 5H2 (43) also engages the DI/DIII linker, but more extensive interaction with DIII, or DI, restricts their specificity to ZIKV and DENV-4, respectively. The highly conserved hinge region between DI and DIII appears to be another site of vulnerability on the flavivirus E protein. This flexible inter-domain linker allows for major structural rearrangements during formation of the fusogenic trimer (76), which makes it an attractive target for the development of new immunogens.

### Quaternary Epitopes

Another group of potent neutralizing antibodies target more complex virion-specific quaternary epitopes consisting of multiple E protomers and are, as such, more likely to be virus specific due to differences in sequence, glycosylation and structural plasticity of E across flaviviruses (**Figure 1**). One example of such antibody is ZIKV-117, a ZIKV-specific DII antibody that cross-links monomers within the E dimers as well as between neighboring dimers, preventing the reorganization of E necessary for viral entry (46). Whereas the antigen binding fragments (Fab) of antibodies binding EDE (31) and DIII (42) engage all 180 E copies on the virion in a 1:1 stoichiometry, ZIKV-117 needs only 60 Fabs to effectively cross-link the glycoprotein shell, contributing to its potent neutralization capacity and its ability to prevent fetal infection and intrauterine fetal demise in mice (45). Similarly, the DENV-3 specific antibody 5J7 is exceptionally potent and able to coat the virus surface with only 60 Fab molecules, with a single Fab binding across the DI-DII hinge, DII and DIII on three different E protomers (47). 5J7 interferes with both attachment and fusion steps, consistent with its multi-domain targeting ability.

## Genetics of ZIKA AND DENGUE Neutralizing Antibodies

Understanding of the antibody repertoire generated by flavivirus infections has been largely shaped by studies aimed at identifying potent therapeutic antibodies in a few select donors and may not generalize to the most prevalent responses. Higher throughput and more systematic antibody characterization (77) is needed to obtain unbiased insights into the B cell responses elicited by flavivirus infection and vaccination. Nonetheless, the most comprehensive study to date has revealed a wide array of VH gene usage with limited overlap between individuals and no association between VH gene and epitope specificity (41). Epitopes such as the fusion loop and the E dimer are targeted by highly divergent antibodies which may make specificity predictions based on gene usage only difficult (27) (**Table 1**). One notable exception is the case of the DIII lateral ridge-targeting antibodies. Recurrent lineages using the VH3-23/VK1-5 combination have been identified in at least eight donors from central and south Americas by four independent groups (33–36). This type of antibodies was further associated with cross-neutralization of DENV-1, suggesting a role of prior DENV-1 immunity in priming those responses. However, one donor did not display serologic evidence of DENV-1 infection (35), indicating that primary ZIKV infection can, in principle, also elicit such lineage. While other specificities have not been studied as extensively, it is likely that other flavivirus-specific public lineages will emerge. A common observation between all these studies is the very low level of somatic hypermutation observed, even among potent neutralizing antibodies (34, 41, 78). A germline-like ZIKV specific neutralizing antibody with no apparent diversification through affinity maturation was identified, indicating that ZIKV can be readily targeted by germline BCRs (79).

**Table 1:** Genetics and characteristics of Zika virus (ZIKV) and dengue virus (DENV) neutralizing monoclonal antibodies.

Antibody	Heavy chain	VH% SHM	Light chain	HCDR3 length	VDJ junction	Epitope	Neutralization potency	Cross neutralization	References
ZIKV-117	VH3-30		VK3-15	12		Quaternary	****	ZIKV specific	(45)
5J7	VH1-69		VK1-39	14	CARDKELLFSRAFDIW	Quaternary	****	DENV-3 specific	(22, 47)
MZ4	VH4-59	5.5	VL1-44	14	CAGLDRYSWNEGGDHW	DI-DIII linker	****	ZIKV, DENV2-3	(20)
Z004	VH3-23		VK1-5	15	CAKDRGPRGVGELFDSW	DIII lateral ridge	****	ZIKV, DENV-1	(34)
Z006	VH3-23		VK1-5	13	CVRDRSNGWSSINLW	DIII lateral ridge	****	ZIKV, DENV-1	(34)
SMZAb5	VH3-23		VK1-5	15	CAKDRSTRGFGELLNYW	DIII lateral ridge	****	ZIKV, DENV-1	(33)
ZIKV-116	VH3-23	6.5	VK1-5	15	CAKDRLSRGVGELYDSW	DIII lateral ridge	***	ZIKV, DENV-1	(36, 45)
1C11	VH3-23	3.5	VK1-5	14	CAKDRIVLGLELFDSW	DIII lateral ridge	**	ZIKV, DENV-1	(35)
ZKA190	VH3-30	2.7	VK3-20	23	CAKSGTQYYDTTGYEYRGLEYFGYW	DIII lateral ridge	***	ZIKV specific	(42)
EDE1-C8	VH3-64D	6.9	VK3-11	15	CVGGYSNFYYYYTMDVW	E dimer	***	ZIKV, DENV1-4	(25, 27)
EDE1-C10	VH1-3	2.8	VL2-14	21	CARDKVDDYGDYWFPTLWYFDYW	E dimer	****	ZIKV, DENV1-4	(25, 27)
EDE2-A11	VH3-74	8.7	VL2-23	26	CVRDGVRFYYDSTGYYPDSFFKYGMDVW	E dimer	**	ZIKV, DENV1-4	(25, 27)
2D22	VH1-69		VL1-47	9	CARRPQSIFDW	E dimer	** (DENV-2)	DENV-2 specific	(22)
MZ20	VH3-11	10.4	VK1-33	10	CVRAGGARIENW	DII bc loop	** (ZIKV)	ZIKV, DENV1-4, JEV	(20)
MZ54	VH3-11	10.4	VK1-33	10	CVCAGGGRTDYW	Fusion loop	**	ZIKV, DENV1-4, WNV	(20)
MZ56	VH3-64	4.9	VL2-11	17	CARGWYYYDSRAYWYFDLW	Fusion loop	**	ZIKV, DENV1-4, WNV	(20)
Z3L1	VH3-30		VL1-51	10	CARDHLGWSSIW	DI, DI-DII hinge	**	ZIKV specific	(21)
1F4	VH3-33		VL1-36	18	CARDKNPGTKPYYHYGMDVW	DI, DI-DII hinge	***	DENV-1 specific	(22, 23)

VH % SHM: % somatic hyper mutation in the heavy chain variable gene. VH % SHM and VDJ junction characteristics were indicated whenever available. Neutralization potency is based on IC50 against ZIKV unless otherwise indicated. IC50 (ng/ml) are indicated as follows: **** (<10); *** (10–50); ** (50–500); * (>500). DIII lateral ridge-directed antibodies sharing heavy and light chain V gene usage are underlined.

## Implications for Vaccine Design and Conclusions

Several neutralizing epitopes on ZIKV and DENV 1-4 have been identified as desirable targets for successful immunogens (17, 20–22, 25, 32–36, 45). In our opinion, the ideal vaccine will be able to elicit protective cross-neutralizing antibodies to ZIKV and DENV 1-4, providing cross-protection while minimizing responses to non-neutralizing epitopes that may result in ADE. Although there are antigenic differences between ZIKV and DENV, and within DENV 1-4, there is evidence that a pan-ZIKV/DENV flavivirus vaccine could be achieved by using different approaches that target the antigenic similarities between these viruses. For example, stabilized E-dimers may be promising candidates as they display EDE, DII, DI-DIII, and DIII neutralization epitopes, which may be exploited by sequential vaccine strategies with different serotypes to guide B cell responses toward conserved ZIKV/DENV cross-neutralizing epitopes (69–71). Further examination is needed on whether they should be used as subunit vaccines or multivalently displayed on scaffold/nanoparticles. Vaccines based on whole virus or virus-like particles are antigenically unique in their ability to present quaternary epitopes capable of eliciting some of the most potent neutralizing antibodies (20, 45), pending various inactivation methods (5, 80). A potential issue with whole inactivated flavivirus vaccines is the residual presence of prM, a target for non-neutralizing antibodies, which would need to be considered in future strategies. However, the development of novel stable cell lines overexpressing furin would allow for the production of fully mature virions, devoid of prM (81), if viral sequences produced within these cell lines are representative of circulating viruses. Recent advances in virus inactivation techniques could further enhance the immunogenicity of such vaccines and allow for fewer immunizations with higher neutralizing antibody titers (82). Finally, among several alternative vaccine platforms, viral-vectored vaccines based on non-replicating adenoviruses that encode for the ZIKV M and E proteins are quickly moving forward. Recent studies revealed that human Ad26, simian RhAd52, ChAd7, and GAd have all been shown to elicit protective responses in animal studies, with rapid and durable neutralizing antibody responses (83–88). It has yet to be determined the neutralizing targets of these protective responses.

Clearly the isolation of highly potent neutralizing antibodies coupled with detailed examination of their properties at the molecular level have provided pivotal insights into the protective targets that can, in turn, inform immunogen design or ultimately a cross-flavivirus vaccine. Until these developments come to fruition, these mAbs offer new options in treatment modalities for flavivirus infections, or as prophylaxis during times of an outbreak to protect populations at risk such as pregnant women in the case of ZIKV or children with severe secondary DENV infections.

## Author Contributions

VD wrote the manuscript, with input and edits by KM and SK. All authors contributed to the article and approved the submitted version.

## Funding

This work was supported by a cooperative agreement (W81XWH-18-2-0040) between the Henry M. Jackson Foundation for the Advancement of Military Medicine, Inc., and the U.S. Department of Defense (DOD). The views expressed are those of the authors and should not be construed to represent the positions of the U.S. Army, the Department of Defense, or HJF.

## Conflict of Interest

The authors declare that the research was conducted in the absence of any commercial or financial relationships that could be construed as a potential conflict of interest.
